# Monetary Reward Modulates Task-Irrelevant Perceptual Learning for Invisible Stimuli

**DOI:** 10.1371/journal.pone.0124009

**Published:** 2015-05-05

**Authors:** David Pascucci, Tommaso Mastropasqua, Massimo Turatto

**Affiliations:** Center for Mind/Brain Sciences, University of Trento, Trento, Italy; University of Gent, BELGIUM

## Abstract

Task Irrelevant Perceptual Learning (TIPL) shows that the brain’s discriminative capacity can improve also for invisible and unattended visual stimuli. It has been hypothesized that this form of “unconscious” neural plasticity is mediated by an endogenous reward mechanism triggered by the correct task performance. Although this result has challenged the mandatory role of attention in perceptual learning, no direct evidence exists of the hypothesized link between target recognition, reward and TIPL. Here, we manipulated the reward value associated with a target to demonstrate the involvement of reinforcement mechanisms in sensory plasticity for invisible inputs. Participants were trained in a central task associated with either high or low monetary incentives, provided only at the end of the experiment, while subliminal stimuli were presented peripherally. Our results showed that high incentive-value targets induced a greater degree of perceptual improvement for the subliminal stimuli, supporting the role of reinforcement mechanisms in TIPL.

## Introduction

Perceptual learning refers to a relative long-term improvement in perceptual tasks following practice. Selective attention and active training on the relevant stimuli have always been considered two fundamental ingredients for perceptual learning to occur [1]. In the last decade, however, the phenomenon of task-irrelevant perceptual learning (TIPL) has challenged this view, showing perceptual improvement for stimuli that are outside the current goal of the observer and that are presented below the sensory threshold [2]. TIPL, however, does not ensue from mere exposure, but is specific for the subliminal stimuli repeatedly paired with the occurrence of task-relevant events [3]. For example, in a typical TIPL paradigm the observer must discriminate a target letter embedded in a rapid stream of distracter letters shown at fixation, while subliminal stimuli are presented peripherally. Crucially, TIPL is found only for the irrelevant stimuli paired with the targets but not for those paired with the distracters [3]. Hence, target processing appears to be fundamental for this form of perceptual learning, and indeed TIPL does not take place if the target remains unnoticed [4]. On the ground of this pattern of results, it has been hypothesized that TIPL occurs because the successful recognition of a task-relevant stimulus triggers an endogenous reward, which functions as an exogenous reward in reinforcement learning [5–7]. Specifically, the reinforcement signal evoked by target detection (or identification) gives rise to the learning of the irrelevant subliminal stimuli that are synchronous with the endogenous reward.

However, although the effects of reward on learning and attention have been widely explored [8–14], clear evidence that TIPL is driven by reward-related reinforcement mechanisms associated with target occurrence is still lacking [15].

To address this issue, we used monetary incentives to manipulate the reinforcing value of target recognition. Participants were given three days of training on a central task associated with either a high or low monetary reward, while subliminal and irrelevant stimuli were presented peripherally. We reasoned that if perceptual improvement is regulated by reinforcement mechanisms, then targets with different reinforcing values should differentially modulate the amount of task-relevant learning and, crucially, of TIPL. Although we manipulated the amount of exogenous reward associated with the target, the reward was not delivered trial by trial on the basis of the correct target identification; rather it was provided only at the end of the experiment as a function of the overall participant’s performance. The reward value (high vs. low) of a given trial was indicated either by the color of the target itself (Experiment 1), or by the color of a cue preceding the target (Experiment 2). This procedure allowed us to achieve two important goals: first, to adopt a paradigm in line with previous TIPL studies in which perceptual improvement for subliminal stimuli has been shown in auto-supervised learning conditions [16]; second, and related to the first point, by omitting the reward on a trial-by-trial basis the process of target identification was made crucial for the activation of the reinforcement system [3], which should be modulated by the level of monetary reward associated with a given target.

## General Method

### Participants

Fourteen (Experiment 1) and twenty (Experiment 2) participants (aged 19–28) with normal or corrected-to-normal vision were recruited from the university population and paid at the end of the of experiment according to their performance. The study was approved by the local institutional ethics committee (Comitato Etico per la Sperimentazione con l’Essere Umano, University of Trento, Italy). Written informed consent was obtained from all participants, and all the experiments were carried out in accordance with the Declaration of Helsinki.

### Apparatus

Participants sat at approximately 60 cm in front of the monitor in a dimly illuminated psychophysics testing room. Stimuli for the test and training phases were generated with Matlab and the Psychophysics Toolbox 3.8 [17] and presented on a gamma calibrated monitor DiamondTron V2-CRT 19” (1024 x 768, 100 Hz). Eye movements were monitored during pre- and post-test sessions with an Eyelink 1000 Tower Mount system (sampling rate: 1000 Hz; SR Research, Ontario, Canada). The apparatus was identical in the two experiments. All experiments were conducted at the Center for Mind/Brain Sciences of the University of Trento, Italy.

### Stimuli and procedure

Each experiment is divided in three phases: a pre-test phase, a training phase, and a post-test phase.

### Test phase

The pre- and post-test phases were used to estimate the participant’s sensitivity to the appearance of a Gabor patch in four possible positions. The Gabor consisted of a sinusoidal grating of 5 cycles/° windowed by a Gaussian envelope of 1°.

Sensitivity in each position was tested separately in different blocks of 200 trials (three blocks in Experiment 1, and four blocks in Experiment 2; each block lasted approximately 10 minutes) with the method of constant stimuli. In each position the Gabor had a different orientation (-60°, -30°, 30° and 60° from vertical), identical to that of the Gabor presented subliminally in the same position during the training phase (see below). The four possible positions corresponded to the corners of an imaginary square centered on the fixation point, with each corner at 3° of retinal eccentricity. The Gabor appeared inside a rectangular (14° x 20°) matrix of dynamic visual noise obtained by redrawing, every 100 ms, 80% of the pixels of the background (mean luminance of 84 cd/m^2^) with random values of luminance (drawn from a uniform discrete distribution). The Gabor was embedded on the noisy background by replacing 80% of its image with the dynamic-noise matrix (see [18] for a similar procedure).

Each trial started with the presentation of a fixation point and the dynamic-noise matrix for 200 ms, followed by two consecutive spatial cues (outlined blue circles of 2°) presented on the position of the Gabor for 100 ms, and separated by a 400-ms interval. The Gabor was presented inside either the 1^st^ or the 2^nd^ spatial cue, with one of five possible contrast levels: 10%, 16%, 26%, 43%, or 70%. Participants were asked to report, in a two-interval forced choice task (2IFC), whether the Gabor appeared inside the 1^st^ or 2^nd^ cue.

### Training phase

**Fig 1:**
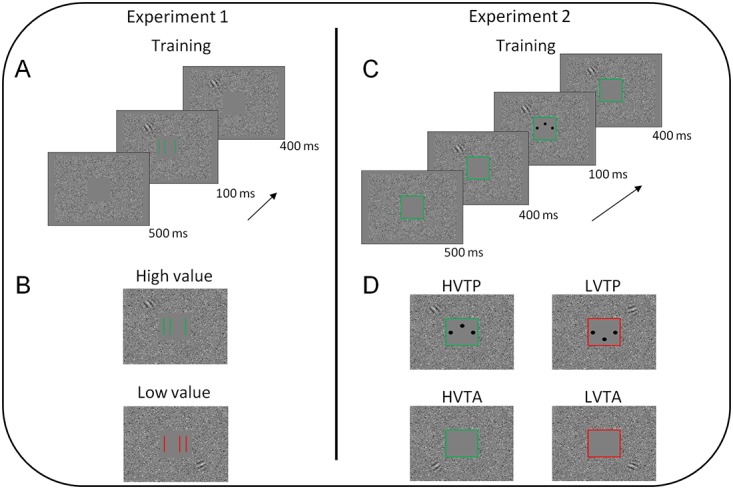
Schematic illustration of a training trial in Experiments 1 and 2. (*A*) Sequence of events in the line-bisection task of Experiment 1: after 500 ms of the dynamic-noise matrix the bisection stimuli and the paired Gabor appeared at the same time; bisection stimuli were presented for 100 ms, while the paired Gabor remained on screen for other 400 ms. (*B*) Example of high and low-value bisection stimuli and the paired irrelevant Gabor presented with different spatial location and orientation. (*C*) Sequence of events in the three-dot hyperacuity task of Experiment 2: after 500 ms of the dynamic-noise matrix the paired Gabor appeared for 900 ms; in the target-present trials, the three dots were presented 400 ms after the onset of the Gabor. The outlined square indicating the high or low-value condition was presented for the entire duration of the trial. (*D*) Example of the four conditions in Experiment 2 and the corresponding irrelevant Gabor presented at four different spatial locations and with a different orientation. The peripheral high-contrast Gabor is depicted in the figures only for representation purpose; the Gabor had a below-threshold contrast (12%) during training.

In the training phase, the background and the dynamic-noise matrix remained the same as in the pre- and post-test phases. However, a small squared region (2° x 2°) centered at fixation was dynamic-noise free and contained, when presented, the training target and the corresponding cue (see Fig 1).

The training target could be of two types. In the line-bisection task, three lines of 1° x 0.1° were presented inside the central noise-free region, with the two flanking lines positioned at 0.7° of eccentricity. The horizontal displacement of the central line was regulated by a 3-down/1-up staircase procedure designed to keep participants accuracy at ~80%. In the three-dot hyperacuity task, three dots (0.14° in diameter) were presented at fixation, and the vertical displacement of the central dot was regulated by the same adaptive procedure.

In Experiment 1, the training target could be either green or red, whereas in Experiment 2 it was always black and anticipated by an incentive cue consisting in an outlined-colored (green or red) square (2° side) (see Fig 1). The training phase was conducted for three consecutive days, with daily sessions of 800 trials divided in 2 blocks (each block lasted approximately 20 minutes). In the first block (line-bisection task) participants were asked to report the lateral offset (Left vs Right) of the central line, while in the second block (three-dot hyperacuity task) they had to report the vertical offset (Up vs Down) of the central dot.

In Experiment 1, each trial started with the presentation of the dynamic-noise area for 500 ms, followed by the appearance of the training target for 100 ms. The crucial manipulation was that together with the onset of the training target a below-threshold Gabor appeared on the screen for 500 ms. The subliminal Gabor appeared in two different positions as a function of the incentive value of the training target, and indicated by the target’s color (green or red). The contrast (12%) of the subliminal Gabor was determined through a 2IFC task in a pilot experiment.

In Experiment 2, each trial started with the presentation of the dynamic-noise area together with a green or red incentive cue, which indicated the value of the upcoming target (if presented) and remained onscreen for the whole trial duration (1400 ms). 500 ms after cue onset, the subliminal Gabor appeared in one of the four positions, and remained on screen for 900 ms. Each position was systematically paired with one of the four experimental conditions, defined by the combination of target presence and target value: High Value Target Present (HVTP), Low Value Target Present (LVTP), High Value Target Absent (HVTA) and Low Value Target Absent (LVTA). In the target-present trials, the target was presented for 100 ms and after 400 ms from the onset of the subliminal Gabor.

At the end of each block of trials participants were reminded of the final monetary reward paired with high- and low-value targets.

### Data analysis

Data from the central task were analyzed by computing the mean of the last five reversals of each staircase procedure and then comparing the averaged means across days and tasks between the two conditions. For the analysis of the pre- and post-test data, the proportion of correct responses of each participant in each condition was fitted to a logistic function of the form:

$$P\left(Y\;=\;1\right)\;=\;\gamma +\left(1-\;\gamma -\lambda \right)\frac{1}{1+exp(-\beta \left(x-\;\alpha \right))}$$

where *P*(*Y* = 1) is the probability of a correct answer, *γ* and *λ* are the guess and lapse rate, *α* is the threshold (or location parameter), *β* is the slope and *x* the contrast level of the Gabor. Parameters of the psychometric function were estimated via maximum likelihood allowing threshold and slope to vary freely and with the only constrains that the guess rate was set at 0.5 and the lapse rate was free to vary within a restricted range of values (0–0.03) [19].

## Experiment 1

In Experiment 1 we measured TIPL for stimuli that were paired with high- and low-value targets during three days of training (Fig 1A). On each daily session of training, participants were asked to perform two visual discrimination tasks at fixation (see General Method). The manipulation of the incentive value of the target in each task was obtained using two different target colors (Fig 1B): correct identification of green target stimuli was valued 40 cents (high-value), whereas correct identification of red target stimuli was valued 4 cents (low-value). High- and low-value targets had the same probability (.5). At the beginning of training, participants were informed that their final payment was estimated on the weighted sum of the total high- and low-value trials in which they responded correctly. During training, one of two irrelevant and below-threshold Gabors, with different orientations and spatial positions (see General Method), was paired with high- and low-value targets respectively. Perceptual improvement in the detection of the irrelevant Gabor paired with either the high- or low-value target was evaluated, before and after training, with a 2IFC (see General Method). In addition, as a control condition we tested perceptual improvement for a new Gabor that was not presented during training. In this control condition, the Gabor had a unique location and orientation, selected from the two that were not paired with high- or low-value targets during training. Locations and orientations were counterbalanced across participants.

### Results

**Fig 2:**
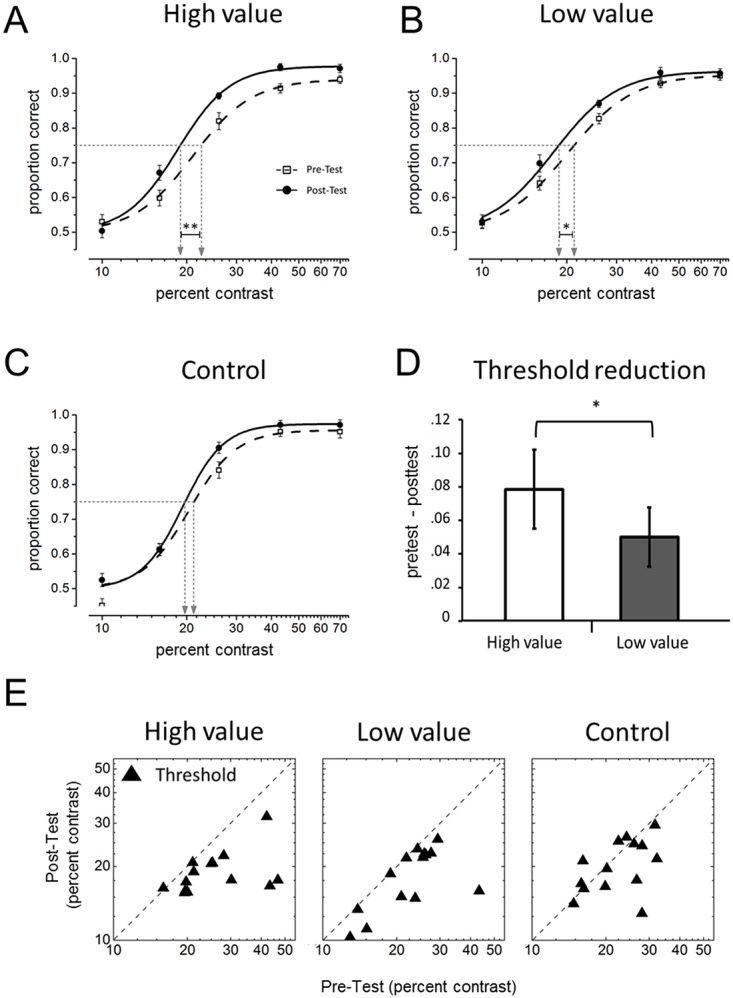
Results from Experiment 1. (*A*,*B*,*C*) Psychometric functions (maximum likelihood logistic function fit) obtained in the 2IFC tasks, plotting the probability of correct responses for the five levels of Gabors contrast (data averaged across participants). The points where the horizontal-dotted line intersects the curves indicate the estimated threshold (at 75% of correct responses). (*D*) Threshold reduction between pre- and post-test for the high- and low-value conditions. (*E*) Pre- and post-test distribution of the participants’ thresholds in the three conditions. The results indicate that the degree of TIPL varied as a function of the target incentive value. * statistical significance at *p* < .05; ** statistical significance at *p* < .01; Error bars represent SEM.

In the training task threshold was lower for target stimuli associated with high rather than low reward (high reward, M = 0.047° ± 0.001; low reward, M = 0.065° ± 0.001; *p* < 0.001, paired *t* test). To calculate the degree of TIPL for the irrelevant Gabors we calculated the participant psychometric functions (pre- and post-test, Fig 2A, 2B and 2C) and analyzed these results with a two-level analysis also defined as Parameter-As-Outcome Model (PAOM; [20]) (see General Method). In the first level of the analysis we fitted the psychometric functions to the proportions of correct responses separately for each participant and condition. In the second level of analysis the individual threshold estimates (Fig 2E) were submitted to a repeated-measure ANOVA with Target value (High, Low and Control) and Test (Pre-Test vs. Post-Test) as factors. The ANOVA revealed a significant two-way Target value x Test interaction *F*(2,26) = 5.78, *p* = 0.008, η_*p*_^2^ = 0.30. Post-hoc analyses showed a contrast threshold reduction between pre- and post-test for the detection of the Gabors paired with high-value targets (Mean reduction = 7% ± 0.02; *p* = 0.006, paired *t* test) and low-value targets (Mean reduction = 5% ± 0.01; *p* = 0.017, paired *t* test), but this effect was not reliable in the control condition (*p* = 0.11, paired *t* test) (Fig 2A,2B,2C and 2E). Importantly, threshold reduction was larger in the high-value than in the low-value condition (*p* = 0.04, paired *t* test), as expected if the internal reinforcement signal that induces TIPL is proportional to the targets incentive value (Fig 2D).

The results of Experiment 1 were in line with those reported by previous TIPL studies showing significant learning effects for subliminal stimuli temporally paired, across multiple days of training, with relevant targets at fixation [3]. To be sure that our Gabors were below threshold during training, in the present and next experiment we used a contrast level (12%) that was smaller than the one (15%) used to estimate the absolute threshold (50% of correct response in a 2IFC task) in the pilot experiment. In addition, at the end the experiment each participant was debriefed as to whether he/she noticed any Gabor in the dynamic noise during training. None of the participants reported to have noticed the Gabor.

The novel finding was to show that the level of delayed-monetary reward associated with the target occurrence modulated the degree of TIPL. This gives strong support to the hypothesis according to which reinforcement mechanisms are responsible for the type of cortical plasticity documented by TIPL.

However, if on the one hand the results of Experiment 1 are in agreement with a role of reward in TIPL, on the other hand it is less clear what type of process associated with target occurrence triggered the reinforcing mechanism leading to TIPL. One possibility is that the strength of the reinforcement signal was determined by the mere recognition of the target feature (here, color) indicating the expected delayed-reward value in the trial, regardless of whether the target task-relevant information was correctly discriminated or not. In other words, targets with higher values may have triggered stronger reinforcement signals only by virtue of their color, which could have acted as a conditioned reward. Alternatively, the reinforcement mechanisms leading to TIPL could have been triggered only by the correct task accomplishment [5].

## Experiment 2

To disentangle between the two possible explanations of the results of Experiment 1, we separated the feature indicating the value of the target from the process of target discrimination. The incentive value of the upcoming target was indicated by a visual cue preceding the appearance of the target. A green-outlined square surrounding the target indicated a high-value trial (40 cents), whereas a red-outlined square indicated a low-value trial (4 cents, Fig 1C and 1D). The target appeared always in black and, crucially, was presented only on half of the trials. The inclusion of target-absent trials allowed us to evaluate whether the appearance of the cued value alone was sufficient to induce TIPL without the contribution of target recognition.

### Results

**Fig 3:**
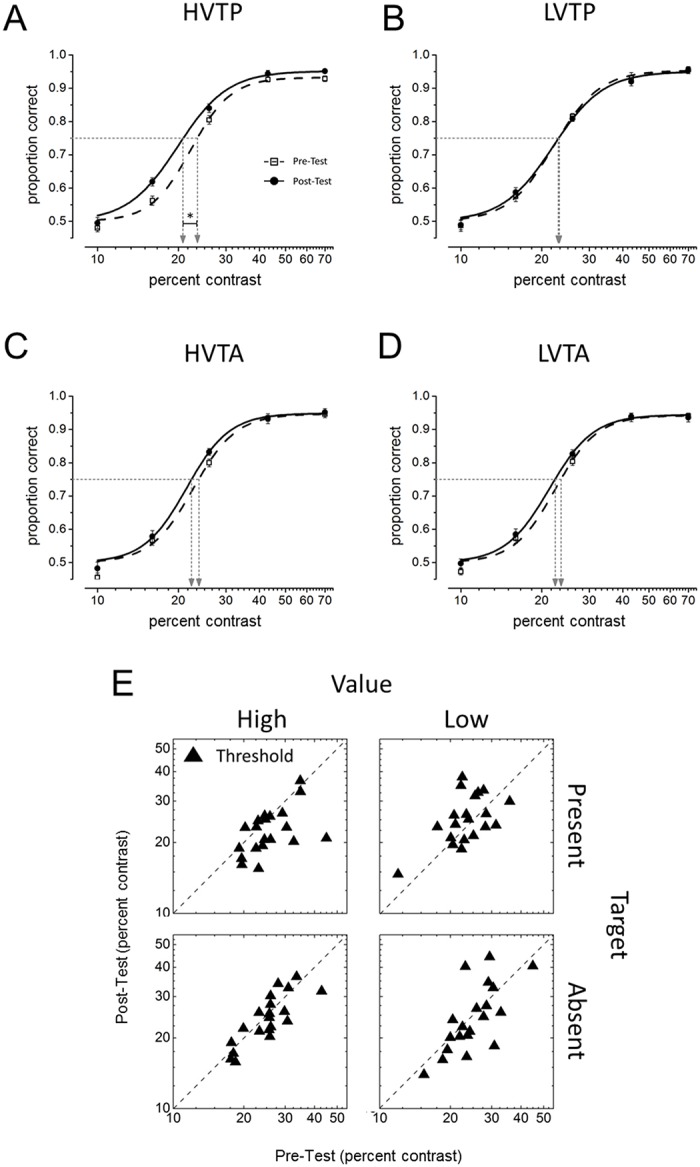
Results from Experiment 2. (*A*,*B*,*C*,*D*) Psychometric functions (maximum likelihood Logistic function fit) obtained in the 2IFC tasks, plotting the probability of a correct response as a function of Gabor contrast (data averaged across participants). The points where the horizontal-dotted line intersects the curves indicate the estimated threshold (at 75% of correct responses). (*E*) Pre- and post-test distribution of the participants’ thresholds in the four conditions. The results indicate that TIPL took place only for high-value targets, but only if the target was presented. * statistical significance at p < .02;. Error bars represent SEM.

As in Experiment 1, participants performed the relevant task with higher precision (i.e. lower thresholds) when the target was presented and was associated with high (HVTP; M = 0.054° ± 0.001) rather than low (LVTP; M = 0.070° ± 0.001) levels of reward (*p* < 0.001, paired t-test). The thresholds estimated with the PAOM procedure (Fig 3) were submitted to a repeated-measure ANOVA with Target (Present vs. Absent), Value (High vs. Low) and Test (Pre-test vs. Post-test) as factors. The ANOVA revealed a significant three-way Target x Value x Test interaction *F*(1, 19) = 4.71, *p* = 0.04, η_*p*_^2^ = 0.20. Post-hoc analyses showed a significant threshold reduction between the pre- and post-test (Mean reduction = 3% ± 0.007) in the HVTP condition (*p* = 0.01, paired *t* test), but no effect of training in the other conditions (Fig 3A,3B,3C and 3D). Hence, in Experiment 2 TIPL occurred only when the irrelevant Gabor was paired with high-value targets. Crucially, no TIPL was found in the HVTA condition, when the cue predicted a high-value target that instead was not presented. This suggests that the mere appearance of a cue indicating a possible high-value target was not sufficient to trigger the reinforcement signal. Therefore, some process related to target detection (or discrimination) was required to the release of reinforcement learning signals, which in turn fostered TIPL.

## General Discussion

Models of TIPL assume that it takes place because the detection of relevant events, such as the target appearance, works as an endogenous reward in the brain, which reacts by releasing diffuse learning signals that strengthen the representation of the subliminal stimuli [5, 6].

In Experiment 1, we found that the amount of learning for task-relevant stimuli was higher when they were paired with high-value targets than with low-value targets. This is in line with theoretical models and previous findings showing that larger reward produces stronger learning [21]. Importantly, the same pattern of results emerged also for the subliminal task-irrelevant stimuli, showing that TIPL varied as a function of the reward magnitude associated with the target. This result provides experimental support to the hypothesized role of reinforcement mechanisms for task-irrelevant subliminal stimuli in auto-supervised tasks.

The idea that reinforcement mechanisms are involved in TIPL has been investigated by Seitz, Kim and Watanabe [22]. In their study, participants were presented with one of two oriented Gabor patches that were below visual threshold, and thus served as subliminal stimuli. One Gabor acted as a conditioned reinforcer for a subsequent rewarding stimulus (a drop of water delivered to the participant’s mouth). Participants were asked to passively observe the stimuli without task. After several days of passive participation the authors found significant perceptual learning for the Gabor paired with the reward. They concluded that reinforcement enhanced the perceptual representation of the subliminal Gabor without the necessity of task-related attention to the stimuli.

While the presence of the final monetary reward makes our study somehow comparable to that of Seitz, Kim and Watanabe [22], we believe the two studies to differ in some important aspects. In Seitz, Kim and Watanabe’s [22] study participants were passively exposed to the conditioned and unconditioned stimuli, whereas we asked participants to perform a central task to receive monetary reward at the end of the experiment. Therefore, in the study of Seitz and colleagues the reinforcement signals leading to TIPL was generated by a pure Pavlovian conditioning, whereas in our study reinforcement was contingent on correct task execution, bringing the procedure closer to operant or Skinnerian conditioning. In relation to this procedural difference, one could argue that despite the fact that the reward was delivered only at the end of the current experiment, the cue acted as a conditioned reinforcer because participants were informed about the contingency between the cue and outcome. However, Experiment 2 showed that TIPL did not appear when there was no central task, thus showing that in our study the reinforcement mechanisms leading to TIPL were yoked to task execution. In other words, TIPL was affected by the target value only if the target was presented and recognized (at least in the majority of trials); when the target did not appear—a condition of passive exposure more similar to the study of Seitz and colleagues—we failed to find evidence of TIPL.

We did not measure reward-related activity in the brain, and because of this one may argue that the current results do not strongly relate to investigations of neural signals evoked by the processing of reward. However the same critique could also be applied to the study of Seitz, Kim and Watanabe [22], and, for that matter, to all the psychophysical studies on TIPL explicitly invoking reinforcement signals triggered by target recognition as the mechanism leading to learning. In addition, even electrophysiological studies directly addressing the response of dopamine neurons to reward acknowledge that the response is driven by salient stimuli (rewards) that alert the organism and attract attention [23], thus showing how difficult is to disentangle the pure effect of reward-induced reinforcement from that of strategic attention [24, 25].

Overall our results are in agreement with the ‘attention-gated reinforcement learning’ model (AGREL) [6], which proposes that learning occurs by trials and errors, and is modulated by the interplay between a global neuromodulatory reward-related signal and selective attention. Attention would gate learning by selectively marking the task-relevant neural representations that need to be potentiated and those that are irrelevant and must be inhibited. The AGREL model describes a form of supervised learning based on external feedback (or reward) given on a trial-by-trial basis, but reinforcement learning can take place also in unsupervised or auto-supervised manner [26, 27]. In the latter case, the reinforcement signal inducing brain plasticity could be triggered on the basis of the observer’s self-estimation that the current task has been accomplished [2], or more in general upon detection of task-relevant events (e.g., the target). A similar mechanism may have operated in our experiments, given that no feedback was provided for the central task; yet, the strength of the reinforcement signal was clearly modulated by the amount of the delayed-monetary reward.

Although the present findings are in agreement and substantiate the hypothesis that TIPL is controlled by reward-related reinforcement mechanisms triggered by target detection (or discrimination), alternative possibilities must be considered and evaluated. For example, one may wonder to what extent attention, rather than reinforcement signals, was the main determinant of TIPL in our experiments. The answer to this question is not straightforward, but depends on the type of attention one refers to. Attention, indeed, is not a unitary process, but consists of at least 3 subsystems: the alerting system, the orienting system, and the executive system [28]. A role of the latter form of attention can reasonably be excluded, as TIPL refers to learning of stimuli that do not require any type of response. As for a possible contribution of spatial or feature-based attention, it could be argued that on high-incentive value trials, when more attention was paid to the central target, more attention was also deployed to the peripheral subliminal stimuli, thus explaining the pattern of TIPL we observed. However, this explanation seems untenable because recent evidence has shown that directing attention to the irrelevant stimuli inhibits, rather than facilitates, TIPL [29]. Thus, orienting and executive attention subsystems do not seem to be involved in inducing TIPL [30].

It is instead entirely possible that attention would be involved in TIPL if one considers the alerting system. According to this view, the appearance of a task-relevant stimulus activates the observer’s alerting system, whose transient activation is accompanied by a diffuse reinforcement signals, possibly mediated by the norepinephrine system, that lead to TIPL [31]. Hence, the non-specific reinforcement signals that control cortical plasticity for irrelevant stimuli can be activated upon target occurrence either because target detection (or discrimination) works as a reward, or because of a boost in the alerting system.

With respect to the latter possibility, the enhanced processing of information paired with the detection of relevant targets has been also reported by recent studies investigating the “attentional boost” effect [32, 33]. In these studies, participants must detect the occasional appearance of a target presented among a stream of distractors, while they simultaneously encode background images for a subsequent memory test. The results show that memory for images presented concurrently with the targets is better than memory for images paired with the distractors. The attentional boost effect assumes that a transient non-specific increase of attention due to target detection leads to enhanced processing of information also in the secondary task, without however postulating any involvement of reward-related mechanisms. Despite the similarities between the effects reported by studies on TIPL and those on attentional boost, it is still unclear to what extent the two phenomena are the same or different, and if they rely on similar brain mechanisms [33].

With regard to our findings, we acknowledge that they are compatible both with the “reinforcement-mechanism” hypothesis, and with the “attention-boost” hypothesis. However, we tend to favor the former because several features of the paradigm we used are typical of TIPL studies rather than of attentional boost studies: first, our participants performed a single task and were not invited to memorize the irrelevant stimuli; second, the irrelevant stimuli were below threshold; and third, the emergence of TIPL required several hours of training.

A further interpretation of the present results could be made in terms of the degree of motivation. For example, it could be hypothesized that the participants’ degree of motivation in the task positively correlated with the task-incentive value, and that this modulated the level of learning observed for both the central and the peripheral stimuli. A strong argument against this account can be made on the basis of the results of Experiment 2, where TIPL was not observed when no target was presented, indicating that the mere motivational state induced by the cue was not sufficient to induce TIPL at all. Hence, although in our paradigm the motivational account can explain the task-relevant learning, it does not seem to be adequate to explain the differing degree of TIPL. The idea that reinforcement signals that lead to TIPL can also explain the perceptual learning for the relevant task strikes us as parsimonious and sufficient.

Finally, one may wonder whether TIPL, at least in our paradigm, was induced by the successful recognition of relevant targets or by an increase in alertness and “vigilance” caused by the onset of the central stimuli (the target elements). According to this latter possibility, it could be argued that the factor modulating TIPL in Experiment 2 was not the correct task execution but the presence or absence of supraliminal onsets during the exposure to irrelevant stimuli. This alternative interpretation entails that any visual onset, including stimuli that are not identified as targets, would evoke TIPL by boosting vigilance and alertness. Although we did not directly address this alternative hypothesis, previous studies have shown that TIPL occurs exclusively when onsets are recognized as targets [4], whereas non-target items, such as alerting signals or visual cues do not induce TIPL [34]. In addition, the fact that TIPL was not observed for supraliminal onsets paired with low monetary reward (in the condition LVTP of Experiment 2) seems to indicate that a mere increase in vigilance evoked by visual onsets cannot systematically evoke TIPL.

To conclude, we have shown that during auto-supervised perceptual learning task TIPL can be modulated by the incentive value of the target, a result that supports the role of reinforcement mechanisms sensitive to reward magnitude in gating visual plasticity for both visible and invisible sensory inputs.

## Supporting Information

S1 Dataset (CSV)

S2 Dataset (CSV)

S1 Readme (RTF)
