# Supplementary material for: Monetary Reward Modulates Task-Irrelevant Perceptual Learning for Invisible Stimuli
Source: PLoS One. 2015 May 5;10(5):e0124009. doi: 10.1371/journal.pone.0124009 (PMC4420259; doi:10.1371/journal.pone.0124009)
Supplement: S1 Readme — (RTF) [file pone.0124009.s003.rtf]

Monetary reward modulates task-irrelevant perceptual learning for invisible stimuli (DATASET)
David Pascucci, Tommaso Mastropasqua and Massimo Turatto
Center for Mind/Brain Sciences, University of Trento, Italy


The datasets ('S1_Dataset.xls' and 'S2_Dataset.xls') contain the relevant variables for the analysis of the pre- and post-test sessions of Experiment 1 and 2, respectively (see General Method in the main text for the fitting procedure).

Data are organized as follows:
HEADER(first row) : 
Obs          = participant's number
Session      = pre- or post-test stage (1=pre-test; 2=post-test)
Cond         = experimental condition
▪	in 'Experiment 1': Cond(1) = High Value / Cond(2) = Low Value / Cond(3) = Control
▪	in 'Experiment 2': Cond(1) = High Value Target Present / Cond(2) = Low Value Target Present 
   			               Cond(3) = High Value Target Absent   / Cond(4) = Low Value Target Absent
RespC             = correct response on each trial (1= correct / 0 = wrong)
TargetInterval = interval of target presentation on each trial (first or second interval)
RespInterval   = interval reported by participants on each trial
RT 		        = reaction times on each trial (although not included or relevant for the reported analysis)
